# Can unvaccinated children be reached through mobile phones? Analyses of national cross-sectional surveys from 70 countries

**DOI:** 10.7189/jogh.15.04232

**Published:** 2025-08-11

**Authors:** Francine S Costa, Thiago M Santos, Larissa AN Silva, Tewodaj Mengistu, Taylor A Holroyd, Daniel R Hogan, Aluisio JD Barros, Cesar G Victora

**Affiliations:** 1 International Center for Equity in Health, Federal University of Pelotas, Pelotas, Brazil; 2 Postgraduate Program in Epidemiology, Federal University of Pelotas, Pelotas, Brazil; 3 Gavi, The Vaccine Alliance, Geneva, Switzerland

## Abstract

**Background:**

Interventions using mobile phones, otherwise known as mHealth interventions, are increasingly being used in low- and middle-income countries to remind families about scheduled child immunisations. Despite this, few studies examined impact on zero-dose children – those who failed to receive a single dose of a routine vaccine. As disparities in mobile phone ownership may limit the effectiveness of mHealth interventions, we assessed associations between mobile phone ownership, gender, wealth, residence, and zero-dose and modelled their potential impact for reaching unvaccinated children.

**Methods:**

We analysed 70 nationally representative surveys with data on immunisations and mobile phone ownership by households and mothers, and correlated ownership with household wealth and place of residence. We performed analyses at the individual child level and pooled across all countries weighted by national populations. We modelled the mHealth interventions’ potential impact on zero-dose prevalence by estimating how many unvaccinated children are reachable *via* mobile phones.

**Results:**

The surveys included 163 527 children aged 12–23 months, with 13.4% being zero-dose. Among them, 34% of mothers and 73% of households had a mobile phone, compared to 60% and 89% for vaccinated children. Mobile phone ownership by mothers ranged from 32% in the poorest to 86% in the wealthiest quintile. A hypothetical 100% effective intervention using household mobile phones would reduce zero-dose prevalence from 13% to 4%, while one using similar effectiveness assumptions for mothers’ phones would reduce national prevalence to 10%. Interventions with effectiveness ranging from 10% to 50% would lead to smaller impact levels. The largest impact is expected in countries like Guinea and Cote d’Ivoire, where both zero-dose prevalence and mobile phone ownership are high.

**Conclusions:**

The potential impact of mHealth interventions for reaching zero-dose children may be limited by mobile phone ownership among mothers and families, particularly among the poor, where we find the greatest number of unvaccinated children.

Immunisation is among the most cost-effective measures for reducing child mortality globally, especially in low- and middle-income countries (LMICs) [1]. Despite documented increases in vaccine coverage since 2000, routine immunisation services still struggle to reach all children, resulting in vaccination coverage that falls short of the World Health Organization's (WHO) Immunization Agenda 2030 (IA2030) target of 90% by 2030 [2]. A challenging aspect of this problem involves reaching unvaccinated children. Being unvaccinated with a diphtheria-pertussis-tetanus (DPT) vaccine is often used as a marker for children who did not receive any routine vaccines, with the terms ‘zero-dose’ and ‘zero-DPT children’ being used interchangeably to describe this population [3]. The IA2030 aims to achieve a 50% reduction in zero-dose children by 2030 compared to 2019 levels [4]. However, in 2023, 14.5 million children worldwide were left without any dose of DPT-containing vaccine, compared to 12.8 million in 2019 [2].

Zero-dose children are particularly common among families with low socioeconomic position [3], those living in rural areas [4] and those with less empowered mothers [5]. A promising strategy for improving immunisation coverage among these children is mobile health, otherwise known as mHealth. It includes the use of mobile devices such as smartphones and tablets within health services, as well as for reaching out to the population with health messages, for example, promotion of vaccinations and reminders for families with children [6,7].

Scientific articles and reports by international organisations frequently highlight that mobile phones have become widely available in LMICs, thus providing a unique channel for delivering health interventions [8]. Success with mHealth, however, will depend on tackling and overcoming the ‘digital divide’, defined as the gap between individuals, households, and regions with differing levels of access to information and communication technologies [9,10].

The use of mHealth to enhance immunisation coverage is an area of considerable research interest, particularly within LMICs. A 2020 study [11] used survey data from 15 LMICs to document important gaps in mobile phone ownership by gender, socioeconomic position, and urban/rural residence. The authors found that mobile phone ownership was associated with improved reproductive, maternal and child health indicators at the household level. This is yet another example of multiple deprivations when a range of disadvantages are clustered at individual, household, and community levels. These multiple deprivations often include poor health, poverty, inadequate nutrition, low education, and lack of basic services. Specifically, lack of child immunisations tends to overlap with low coverage of other maternal and child interventions, as well as with poverty, low education, poor nutrition, and lack of water and sanitation [12,13]. Given that lack of access to digital technology is associated with poor health conditions and social deprivation, it is important to investigate how likely digital interventions are to reach those individuals, households, and communities suffering from the highest disease burden.

A recent umbrella review [14] examined 62 systematic reviews on multiple types of child immunisation interventions and found that caregiver-oriented strategies, such as sending written and pictorial messages *via* SMS or flyers, led to a pooled improvement of 24% in coverage rates. However, most of the studies identified by the reviewers focussed on follow-up reminders for parents of children late in receiving their shots. The umbrella review only found four studies on zero-dose children, which showed inconclusive results.

A 2022 scoping review [7] explored mobile phone interventions like voice calls and SMS reminders that targeted caregivers in LMICs. It found that SMS reminders sent shortly before vaccination days led to an average increase of about 10 percentage points (pp) in immunisation coverage. Another systematic review published in 2021 [15] compared the effectiveness of various communications strategies and found that combining voice messages with SMS reminders was more effective than phone calls alone. In contrast, standalone SMS reminders showed no significant impact. Evidence from single-country studies from Indonesia, Vietnam, Nigeria, and Cote d’Ivoire suggests increases of around 10–50% in vaccination when reminders were sent to families of children who had received an earlier dose [16–19].

A more recent systematic review focussed on African studies utilising mHealth, both alone and in conjunction with financial incentives, for improving vaccine coverage [6]. It showed a significant increase in coverage with a pooled odds ratio of 2.15. However, odds ratios tend to exaggerate coverage ratios. For example, taking the typical current coverage levels for sub-Saharan Africa, an odds ratio of 2.15 would approximately correspond to an increase from 65% to 80% in vaccination coverage among children reachable through a mobile phone. This translates to a 23% relative increase or 15 pp. A 2025 scoping review confirmed that most studies showed improvements in coverage because of mHealth reminders but were unable to provide quantitative summaries of the impact [20]. Taken together, these findings suggest that the impact of mHealth interventions typically ranges from 25% to 50%.

Here we examine the extent to which mHealth interventions could help reach zero-dose children with vaccination in 70 LMICs based on data on current vaccine coverage and ownership of mobile phones. First, we describe the proportions of mothers and households who own mobile phones in these countries and how these proportions vary by wealth and urban/rural residence. Second, we show how phone ownership varies according to the zero-dose status of the children. Lastly, we simulate the potential impact on zero-dose prevalence and related inequalities by using cell phones to deliver messages to mothers and families of unvaccinated children, and by assuming different scenarios of how effective such cell phone messages might be in leading these children to be vaccinated.

## METHODS

Our methods and findings are reported according to the STROBE guidelines (Checklist S1 in the **Online Supplementary Document**). The surveys included in our analyses were selected from the global repository maintained by the International Center for Equity in Health, which includes almost 460 publicly available, nationally representative surveys from 120 countries [21]. This analysis relied upon 70 Demographic and Health Surveys (DHSs) [22] and Multiple Indicator Cluster Surveys (MICSs) [23] containing information on mobile phone ownership and child vaccinations. We used the most recent eligible survey per country, carried out in 2015 or later.

The outcome was zero-dose prevalence, or the proportions of surveyed children aged 12–23 months who failed to receive any doses of a DPT-containing vaccine [24]. During data collection, when a home-based record with vaccination information was not available for inspection, the survey respondent’s report on vaccinations was recorded. Accordingly, both maternal recall and home-based records were used to generate the zero-dose indicator, following WHO recommendations [25]. Missing values were treated as a lack of vaccination, as is standard practice in coverage analyses [25]. Table S1 in the **Online Supplementary Document** provides information by country on the sources of information on immunisation status and on missing values. Information from the women’s and household DHS questionnaires allowed for assessing mobile phones in the household and phones owned by children's mothers. Mothers’ phones are also considered as phones available in the household, but due to data inconsistencies, 1.7% (median value for all countries) of the households reportedly did not have a phone when the mother reported having one.

Children were also characterised according to household wealth quintiles and urban-rural residence. Quintiles were based on wealth indices provided in the DHS datasets, which are derived through principal component analyses of household assets (presence of radio, television, refrigerator, *etc*.) and characteristics of the homes, including the availability of electricity, water supply, and sanitary facilities [26]. Since relevant assets may differ between urban and rural households, the wealth index is initially calculated separately for each area and later combined into a single national index by using a scaling procedure to ensure comparability between the area scores. The area of residence is categorised as urban or rural, depending on country-specific delimitations [26]. Sex of the child was also used as a stratification variable.

We expressed wealth-related inequalities using the slope index of inequality (SII), which represents the beta parameter of a logistic regression with zero-dose prevalence as the dependent variable and wealth quintiles as the independent variable. We multiplied the resulting beta values by 100, indicating the prevalence gap in pp among children in the wealthiest and poorest households. An SII value of zero represents complete equality, with negative values showing that zero-dose prevalence was inversely proportional to wealth and positive values showing direct associations. Inequality by place of residence was expressed as the difference in prevalence between rural and urban areas.

The analyses included an examination of mobile phone ownership by wealth and residence, followed by zero-dose prevalence by mobile phone ownership, wealth, and residence. We performed all statistical analyses at the individual level using *R*, version 4.3.1 (R Foundation for Statistical Computing, Vienna, Austria) and Stata, version 18 (StataCorp LLC, College Station, Texas, USA), accounting for the multi-stage survey design. In the pooled analysis of the 70 countries, we calculated zero-dose prevalence according to explanatory variables using logistic regression and the ‘margins’ command in Stata, including fixed effects for each country. We weighted the pooled estimates by national populations of children aged 12–23 months in 2019 (the median year of the surveys).

We used simulation analyses to estimate the potential impact on zero-dose prevalence of digital interventions using mobile phones under five hypothetical impact scenarios: 0%, 10%, 25%, 50%, and a hypothetical 100% effectiveness levels. The first (0%) scenario indicated that the intervention had no effect. In contrast, the last (100%) scenario meant that the intervention would result in every contact through a mobile phone leading to the child being immunised. We estimated national post-intervention zero-dose prevalence from baseline national zero-dose prevalence minus the product of postulated intervention effectiveness times the proportion of zero-dose children with a mobile phone in the household, considering the intermediate scenarios (10%, 25%, and 50% effectiveness). We repeated the simulation exercise for children whose mothers had a mobile phone and also conducted it at the aggregate level for each subpopulation, *i.e.* by wealth quintiles and urban/rural.

### Ethical aspects

The national institutions that conducted the surveys obtained ethical approval, while our analyses were based on anonymised datasets.

### Role of the funding source

Authors TAH, TM, and DRH are employed by Gavi, the Vaccine Alliance, the study funder. All three authors participated in the study design, interpretation of results, and writing up of the manuscript.

## RESULTS

The total sample comprised 163 527 children from 70 countries, ranging from 114 children in Tuvalu to 43 436 in India (median = 1600, interquartile range = 860–2215). The regions with the highest representation were West & Central Africa (83.3%) and South Asia (75.0%), followed by Eastern & Southern Africa (64.0%), East Asia & the Pacific (39.4%), Middle East & North Africa (31.6%), Eastern Europe & Central Asia (19.0%), and Latin America & the Caribbean (13.5%). The study sample included 71.0% of all low-income countries, 61.5% of all lower-middle-income countries, and 29.1% of all upper-middle-income countries worldwide.

Regarding ownership of mobile phones and considering the total weighted sample, 87% of households had a mobile phone, while 56% of the mothers owned one (Figure S1 in the **Online Supplementary Document**). In the total weighted sample, 87% of households had a mobile phone, while 56% of the mothers owned one. We observed the widest gap in Turkmenistan, where 100% of households had a mobile phone, but only 41% of mothers had one. In countries with high phone coverage, such as the Maldives, Armenia, Tonga, Vietnam, and Suriname, these gaps were narrow, as more than 90% of the mothers had a phone. The lowest ownership levels were observed in the Central African Republic, the Democratic Republic of Congo, Burundi, Madagascar, Malawi, Ethiopia, and Papua New Guinea, where fewer than 60% of households and 30% of mothers owned phones. Missing values for DPT vaccination were below 6% when considering the pooled estimate for all countries, even though some countries had values higher than 20%, namely Kiribati (40.1%), Yemen (30.9%), the Democratic Republic of the Congo (32.1%), Nigeria (27.2%), Lao PDR (22.0%), and Samoa (21.9%).

**Figure 1:**
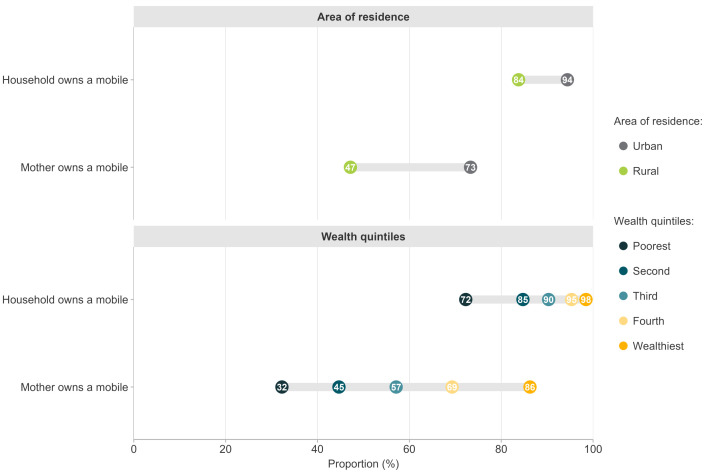
Proportion of households and mothers with a mobile phone according to wealth quintiles and area of residence. Pooled analyses of 70 countries.

In the pooled individual-level analyses of the 70 countries, the availability of mobile phones varied markedly according to wealth and place of residence (**Figure 1**). In the poorest quintile, 32% of mothers and 72% of households had a phone, compared to 86% and 98%, respectively, in the wealthiest quintile. Ownership was 47% among rural and 73% among urban mothers, whereas the corresponding gap for household phones was from 84% to 94%. These results did not vary according to sex of the child (Figure S2 in the **Online Supplementary Document**).

Zero-dose prevalence in the pooled analyses was 13.4% (95% confidence interval (CI) = 13.0–13.8). The highest prevalence was observed when neither the mother nor the household had a mobile phone (25.3%; 95% CI = 23.9–26.8), followed by households with a phone where the mother did not have her own phone (19.2%; 95% CI = 18.3–20.0). The lowest prevalence was observed when mothers had their own phones (10.0%; 95% CI = 9.5–10.6). Adjustment for residence and wealth did not significantly change these findings.

**Figure 2:**
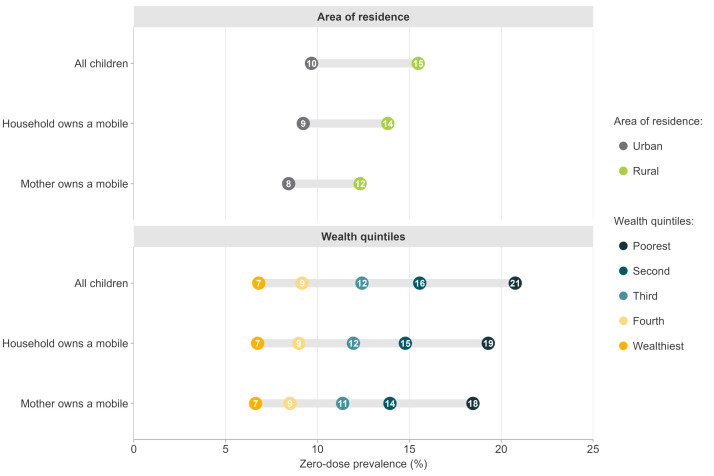
Zero-dose prevalence according to place of residence and wealth quintiles for all children, for children in households with a mobile phone, and for children whose mother owns a mobile phone. Pooled analyses of 70 countries. All *P*-values <0.001.

Prevalence was 5.7 pp higher in rural areas than in urban areas (**Figure 2**; Table S2 in the **Online Supplementary Document**). The gaps were somewhat narrower when a mobile phone was present in the household (4.6 pp) or owned by the mother (3.9 pp). We observed wide zero-dose prevalence gaps between the wealthiest and poorest quintiles – 13.9 pp in the overall sample, 12.6 pp in households with a mobile phone, and 11.9 pp when the mother owned a phone. For children from the poorest quintile, zero-dose prevalence levels were 20.7%, 19.3%, and 18.5%, respectively, whereas for children from the richest quintile, prevalence was similar regardless of mobile phone ownership. The results were similar for girls and boys (Figure S3 in the **Online Supplementary Document**).

In the pooled analyses of 70 countries, 34% of the mothers of zero-dose children had a mobile, compared to 60% of the mothers of vaccinated children. Therefore, 66% of the mothers of unvaccinated children (100% to 34%) could not be reached through their own phones. Household phones were available for 73% of unvaccinated and 89% of vaccinated children, meaning that 27% of zero-dose children could not be reached in this way. In virtually all countries, associations with zero-dose were stronger for mother’s phones than for household phones (Figure S4 in the **Online Supplementary Document** ).

Still, these proportions vary widely from country to country (Figure S4 in the **Online Supplementary Document**), with low-income countries, particularly those in Sub-Saharan Africa, showing the lowest phone ownership levels and the widest gaps among zero-dose and vaccinated children (Table S1 in the **Online Supplementary Document**).

**Figure 3:**
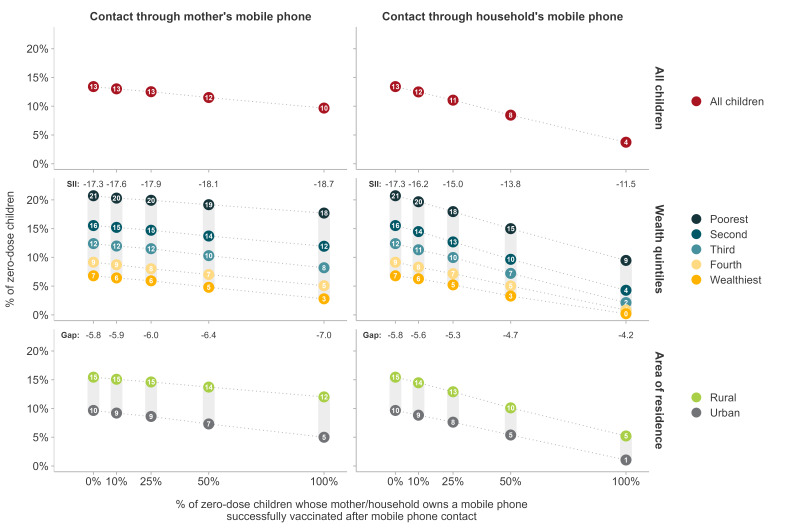
Simulation analyses of zero-dose prevalence (national, by wealth quintiles, and by place of residence) of the potential impact of promoting immunisations through mobile phones under five effectiveness scenarios. SII – slope index of inequality according to wealth.

Next, we estimated the potential impact of promoting immunisations through mobile phones. Based on the pooled sample from the 70 countries, five scenarios were presented with increasing hypothetical effectiveness levels of the promotion intervention, ranging from 0%, indicating a lack of any intervention (or an intervention without any effect), up to 100% representing a hypothetical scenario in which all zero-dose children whose mother or household has a mobile phone were successfully vaccinated (**Figure 3** ). Starting with the current pooled zero-dose prevalence of 13%, contacts through the mother’s phones might reduce prevalence by 3 pp even for an intervention with hypothetical 100% effectiveness, while contact using phones in the household would reduce prevalence by 9 pp. More credible levels of effectiveness (from 10% to 50%) would lead to markedly less impact (**Figure 3**).

We also estimated impact levels after stratification by wealth and residence (**Figure 3**). Interventions delivered through phones owned by mothers would not reduce wealth-related inequalities, with SII values remaining close to 17 pp in the five effectiveness scenarios. The rural-urban gaps would also remain unchanged at around 6 pp. In contrast, effective interventions delivered through household phones would be expected to reduce inequalities according to wealth and place of residence. These results (**Figure 3**) are explained by mothers’ lower levels of mobile phone ownership relative to the number of phones available in household (**Figure 1**). In particular, zero-dose children are concentrated in the poorest quintile, where few mothers own a phone.

The final simulations are restricted to household phones as their potential is greater than for mothers’ phones (**Figure 3**). There was a moderate inverse ecological correlation (Pearson coefficient of −0.44) between national zero-dose prevalence and the presence of mobile phones in households with zero-dose children. Countries with high zero-dose prevalence combined with low phone ownership were mainly located in sub-Saharan Africa, along with a few other low and lower-middle-income countries. Few countries had high zero-dose prevalence and high mobile phone ownership (Figure S5 in the **Online Supplementary Document**). We identified potential absolute reductions from mHealth interventions by country (**Table 1**). The largest absolute reductions, assuming 25% effectiveness of an intervention targeted to household mobile phones, would be expected in Guinea (8.5 pp) and Cote d’Ivoire (7.1 pp) due to the above-mentioned combination of high zero-dose prevalence and frequent mobile ownership. Another 22 countries would have 3 pp or greater reductions, while the remaining 48 countries would have smaller absolute reductions.

**Table 1:** Simulation analyses of the potential impact of promoting immunisations through mobile phones by country, ranked by absolute reduction levels in pp

						% of post-intervention national zero-dose prevalence according to postulated impact levels				
**Country**	**% of baseline national zero-dose prevalence**	**% of zero-dose children with a phone in the household**		**10%**	**25%**		**50%**	**100%**		**Absolute (pp) reduction in national zero-dose prevalence with 25% effectiveness**
Guinea	37.7	89.9		34.3	29.2		20.7	3.8		8.5
Cote d’Ivoire	30.0	95.1		27.2	22.9		15.8	1.5		7.1
Kiribati	40.1	70.3		37.3	33.1		26.0	11.9		7.1
Yemen	32.5	86.7		29.7	25.4		18.4	4.3		7.0
Afghanistan	35.7	74.6		33.0	29.0		22.4	9.1		6.7
Samoa	29.5	88.6		26.9	23.0		16.4	3.4		6.5
Lao	27.1	85.5		24.8	21.3		15.5	3.9		5.8
Nigeria	29.7	76.6		27.4	24.0		18.3	6.9		5.7
Timor-Leste	21.6	86.3		19.7	16.9		12.3	2.9		4.7
Suriname	19.7	94.5		17.9	15.1		10.4	1.1		4.7
Comoros	19.6	90.3		17.9	15.2		10.8	1.9		4.4
Papua New Guinea	36.1	46.0		34.4	32.0		27.8	19.5		4.2
Gabon	16.4	92.1		14.9	12.6		8.8	1.3		3.8
Benin	19.5	74.9		18.1	15.9		12.2	4.9		3.7
Mali	17.9	81.4		16.5	14.3		10.6	3.3		3.7
Niger	19.4	73.0		18.0	15.9		12.3	5.2		3.5
Angola	31.2	43.4		29.9	27.8		24.5	17.7		3.4
Ethiopia	25.7	52.4		24.4	22.4		19.0	12.2		3.4
Iraq	13.3	98.0		12.0	10.1		6.8	0.3		3.3
Mozambique	23.9	53.5		22.7	20.7		17.5	11.1		3.2
Cameroon	16.7	74.9		15.5	13.6		10.4	4.2		3.1
Pakistan	13.7	89.1		12.4	10.6		7.6	1.5		3.0
Philippines	13.3	85.2		12.2	10.5		7.7	2.0		2.8
Democratic Republic of the Congo	34.1	33.3		32.9	31.2		28.4	22.7		2.8
Haiti	16.5	63.2		15.4	13.9		11.3	6.1		2.6
Mauritania	12.2	84.3		11.1	9.6		7.0	1.9		2.6
Indonesia	11.1	88.0		10.2	8.7		6.2	1.3		2.5
Maldives	9.2	100.0		8.3	6.9		4.6	0.0		2.3
CAR	45.0	20.1		44.1	42.7		40.5	35.9		2.3
Kyrgyzstan	9.4	95.9		8.5	7.1		4.9	0.4		2.2
South Africa	8.8	93.3		8.0	6.8		4.7	0.6		2.1
Madagascar	21.7	36.5		21.0	19.8		17.8	13.8		2.0
Togo	9.2	84.3		8.4	7.2		5.3	1.4		1.9
Dominican Republic	8.1	92.9		7.3	6.2		4.3	0.6		1.9
Tajikistan	7.6	97.5		6.9	5.8		3.9	0.2		1.9
Lesotho	8.5	84.8		7.7	6.7		4.9	1.3		1.8
Jordan	7.4	96.6		6.7	5.6		3.8	0.3		1.8
Guinea Bissau	7.0	95.9		6.3	5.3		3.6	0.3		1.7
Cambodia	7.7	86.9		7.0	6.0		4.3	1.0		1.7
Guyana	8.8	71.8		8.2	7.2		5.6	2.5		1.6
Eswatini	6.3	97.3		5.7	4.8		3.3	0.2		1.5
India	6.4	94.1		5.8	4.9		3.4	0.4		1.5
Liberia	8.6	59.0		8.0	7.3		6.0	3.5		1.3
Burkina Faso	5.1	98.8		4.6	3.8		2.6	0.1		1.3
Vietnam	4.9	95.2		4.4	3.7		2.6	0.2		1.2
Nepal	5.3	87.3		4.8	4.2		3.0	0.7		1.2
Tunisia	4.6	100.0		4.1	3.5		2.3	0.0		1.2
Algeria	4.5	97.2		4.1	3.4		2.3	0.1		1.1
Zimbabwe	5.5	79.2		5.1	4.4		3.3	1.1		1.1
Tanzania	5.4	80.4		5.0	4.3		3.2	1.1		1.1
State of Palestine	4.5	95.4		4.1	3.4		2.3	0.2		1.1
Sierra Leone	5.4	70.8		5.0	4.4		3.5	1.6		1.0
Senegal	3.8	99.0		3.4	2.9		1.9	0.0		1.0
Tonga	3.6	100.0		3.3	2.7		1.8	0.0		0.9
Uganda	5.1	69.6		4.7	4.2		3.3	1.5		0.9
Fiji	3.3	100.0		3.0	2.5		1.7	0.0		0.8
Ghana	2.9	92.7		2.7	2.3		1.6	0.2		0.7
Kenya	2.9	91.6		2.6	2.2		1.6	0.2		0.7
Malawi	4.6	41.9		4.4	4.1		3.6	2.7		0.5
Gambia	1.7	100.0		1.5	1.2		0.8	0.0		0.4
Armenia	1.5	100.0		1.4	1.2		0.8	0.0		0.4
Mongolia	3.0	51.8		2.8	2.6		2.2	1.4		0.4
Sao Tome and Principe	2.3	66.4		2.1	1.9		1.5	0.8		0.4
Bangladesh	1.5	100.0		1.4	1.1		0.8	0.0		0.4
Cuba	2.6	57.6		2.4	2.2		1.8	1.1		0.4
Zambia	2.1	51.8		2.0	1.8		1.6	1.0		0.3
Tuvalu	1.7	55.6		1.6	1.5		1.3	0.8		0.2
Turkmenistan	0.7	100		0.6	0.5		0.4	0.0		0.2
Rwanda	0.4	63.7		0.4	0.3		0.3	0.1		0.1
Burundi	0.8	27.9		0.8	0.7		0.7	0.6		0.1

pp – percentage point

## DISCUSSION

While the evidence base is still growing, studies have reported effective interventions for increasing vaccination coverage through mHealth interventions [14,15]. However, few studies have looked at mHealth interventions designed specifically to reach zero-dose children, who may lack contact with the health system [12,13]. Our analysis looked at associations between zero-dose prevalence and mobile phone ownership across key equity stratifiers to understand the potential for mobile-phone-based interventions to increase vaccination coverage in children who miss out on receiving any vaccination.

Although mobile phones are increasingly common in LMICs, like access to immunisation, their ownership is not equitably distributed. Our results confirmed earlier analyses showing that gender, wealth, and place of residence are strongly associated with the availability of such phones [11,27]. In our pooled analyses, 56% of the women had their own mobile, compared to 87% of the households. The finding that phones are available in almost nine out of ten households is impressive, but the pooled proportions ranged from 72% to 98%, from the poorest to the wealthiest quintile. The gap was wider for mothers’ phones, ranging from 32% to 86%. The urban-rural gap was also marked, albeit narrower than the wealth-related gap. While gender gaps likely reflect cultural issues such as women’s empowerment, socioeconomic position affects the ability to purchase a phone and to afford airtime, whereas place of residence may limit the availability of networks.

With respect to potential mHealth interventions, zero-dose prevalence was almost three times higher when neither the mother nor the household had phones, compared to when both had phones. Within the same wealth quintile or place of residence, zero-dose children were more likely to be present when phones were absent. Therefore, reaching a substantial number of zero-dose children through mHealth campaigns could be challenging.

Bearing such inequalities in mind, we simulated the potential impact of mHealth for reaching families with unvaccinated children. Expected reductions in national zero-dose prevalence were computed as the proportion of unvaccinated children whose mother or family would be reachable by mobile phones times the effectiveness of the intervention, that is, the proportion of unvaccinated children who would likely be immunised as a result of the intervention. Although relative reductions do not depend directly on national prevalence before the intervention, absolute reductions in unvaccinated children will tend to be larger in high-prevalence countries.

In the pooled simulation analyses, household phone interventions could reduce prevalence from 13% to 10% with 25% effectiveness and to 8% with an assumption of 50% effectiveness. Because mothers are the primary caregivers and often primary health care decision-makers, and because zero-dose prevalence is more closely associated with women’s than with household phones ownership, one might expect mHealth messages to have a greater impact when delivered to phones owned by mothers. Possible reasons for this expectation include increased access to health-related information, improved communication with health care providers, and greater autonomy in health decision-making among mothers with mobile phones. Yet, the simulated effects were smaller when mothers were targeted and, in addition, had smaller potential effects on reducing inequalities in zero-dose prevalence. This paradox derives from the higher prevalence of household phones and their more equitable distribution in the population than observed for women-owned phones, thus making it harder to reach the most vulnerable children through the latter. An important finding of the analyses is heterogeneity from country to country in terms of mobile ownership levels, gender gaps in ownership, and their associations with zero-dose (*e.g.* in **Table 1** and in Figures S4 and S5 in the **Online Supplementary Document**). The intersectionality among these three variables suggest that decisions on the potential of mHealth interventions need to be tailored to specific country scenarios. For example, impact in Latin America, where cell phone ownership is very high and zero-dose prevalence low, will likely be very different from that in most countries in sub-Saharan Africa, where ownership is lower and zero-dose children are more prevalent.

Our simulations show that the largest absolute impacts on zero-dose prevalence are expected in countries such as Guinea and Cote d’Ivoire, where both zero-dose prevalence and mobile phone ownership are high. The simulation exercises have at least three caveats. The first is the assumption that messages delivered to a person in the household (not always to the mother) would be equally effective as messages received directly by the mother. Second, when dialling a registered mobile number, health workers would seldom know whether the phone belongs to the mother, nor necessarily whether the child has been vaccinated – for example, the phone number may have been recorded soon after birth. Lastly, our equity modelling approach assumes that the likelihood of a child being successfully immunised due to the intervention is random, which may not be the case if socioeconomic position or place of residence affects uptake and compliance with advice on immunisation. Furthermore, mobile phone ownership does not necessarily mean that individuals and households are reachable. In a study conducted in Brazil, nearly half of the low-income households with a known phone could not be contacted 18 months after the initial survey [28], most likely due to payment defaults or frequent changes of mobile phone numbers due to provider price wars. This situation is likely to be affected by low female empowerment, access to funds, and to household dynamics.

The strengths of our analyses include the national representativeness of the survey samples, the use of standardised questionnaires, indicators, and variable definitions, and uniform statistical methods in the analyses. Ours was the largest multicountry analysis relating mobile phone ownership to sociodemographic and immunisation patterns, covering 70 of the approximately 130 LMICs.

Limitations include the cross-sectional nature of the data, that information was only available for 70 of 130 LMICs worldwide, and that the median date of these surveys was 2019. Based on a limited number of countries, it is estimated that the gender gap in mobile internet usage fell from 21 pp in 2019 to 15 pp in 2023 but still remains above 30 pp in sub-Saharan Africa and South Asia [10]. The analyses of mobile phone ownership by wealth quintiles should be approached with caution, as mobile phones are part of the assets used to estimate the wealth index in many countries, which can create a tautological relationship. Despite this concern, the potential bias is likely minimal, as phones represent only one of 25–30 assets included in the wealth index. Unpublished analyses conducted at the International Center for Equity in Health that included 34 DHS and MICS found that mobile phones, on average, ranked 10th among the 27 or so assets frequently included in the index. The principal component analysis showed an average loading of 0.11 for mobile phones (Vidaletti and Barros, personal communication, 24 September 2024). Thus, it is unlikely that such bias would explain the difference of over 50 percent points in woman’s phone ownership between the wealthiest and poorest quintile.

With these caveats and limitations in mind, given that our results suggest that delivering an intervention through household phones could have a greater potential impact on coverage levels and inequalities, the design of mHealth interventions for immunisation should account for a possible need for their delivery through household-owned phones.

Practical considerations affect how our simulation findings may be applied in real life. When a phone number is available for a child in facility records, health workers seldom know whether it belongs to the mother or to someone else in the household, so any available phones will have to be contacted. Because unvaccinated children and their mothers are also less likely to visit health services for other types of care [13], they may not be included in facility registries even when a phone is available in the household. This suggests that strategies for reaching out to all available phones in a geographic area, such as SMS mass outreach campaigns, may achieve higher coverage than attempting to reach phones recorded in a health services registry. More research is needed on these barriers, particularly on how households without any phones may or may not be effectively reached by sensitisation and education campaigns [14] – *e.g.* using local radio or television. It is essential that new research on this topic includes equity-relevant stratified analyses.

## CONCLUSIONS

Existing literature suggests 25% to 50% effectiveness for mHealth reminders on timely vaccination [6,7,14,15], although there is limited evidence of impact on reaching zero-dose children [14]. Our analysis suggests patterns of phone ownership could make it challenging to reach zero-dose children. However, mobile phone ownership is rapidly changing and expanding, so future assessments such as ours may have more promising results on the potential impact of mHealth on reaching children who currently miss out on any immunisation. Ownership of phones by women is closely related to empowerment levels (Table S4 in the **Online Supplementary Document**) [29], so initiatives outside the health sector – for example, removing economic and cultural barriers to providing all women with mobile phones – will be important on their own and potentially contribute to mHealth interventions in general.

## Additional material


Online Supplementary Document
