# Supplementary material for: Can unvaccinated children be reached through mobile phones? Analyses of national cross-sectional surveys from 70 countries
Source: J Glob Health. 2025 Aug 11;15:04232. doi: 10.7189/jogh.15.04232 (PMC12336926; doi:10.7189/jogh.15.04232)
Supplement: Online Supplementary Document [file jogh-15-04232-s001.pdf]

Supplement to: Costa FS, Santos TM, Silva LAN, Mengistu T, Holroyd TA, Hogan DR, Barros AJD, Victora CG. Can unvaccinated children be reached through mobile phones? Analyses of national cross-sectional surveys from 70 countries. J Glob Health. 2025;15:04232.

Table S1. List of countries, survey years, number of children aged 12-23 months, proportions of households and mothers with a mobile phone, and zero-dose prevalence.

Country (ISO) and year	Source	Number of children[1]	Any mobile phones in the household % (95%CI)	Mother owns mobile phone % (95%CI)[3]	Zero-dose % (95%CI)	Missing data on DTP[2] % (95%CI)
**East Asia & the Pacific**		**16001**	**93.4 (92.7-94.0)**	**81.9 (80.7-82.9)**	**11.3 (10.4-12.3)**	**0.8 (0.6-0.9)**
Fiji (FJI) 2021	MICS	417	93.7 (90.8-95.8)	79.9 (75.6-83.6)	3.3 (1.9-5.7)	2.9 (1.6-5.2)
Indonesia (IDN) 2017	DHS	3534	94.0 (92.8-94.9)	79.1 (77.3-80.8)	11.1 (9.8-12.7)	0.4 (0.2-0.7)
Cambodia (KHM) 2021	DHS	1687	94.8 (93.3-96.0)	82.4 (79.7-84.7)	7.7 (6.2-9.4)	0.8 (0.4-1.4)
Kiribati (KIR) 2018	MICS	453	72.1 (67.4-76.5)	50.8 (45.5-56.0)	40.1 (35.1-45.4)	40.1 (35.1-45.4)
Lao (LAO) 2017	MICS	2215	89.8 (88.0-91.4)	65.8 (62.7-68.8)	27.1 (24.7-29.7)	22.0 (19.7-24.5)
Mongolia (MNG) 2018	MICS	1077	98.8 (98.0-99.3)	96.9 (95.4-97.8)	3.0 (1.9-4.5)	1.1 (0.6-2.0)
Philippines (PHL) 2022	DHS	1576	93.6 (91.9-94.9)	86.3 (83.8-88.5)	13.3 (11.3-15.7)	0.3 (0.1-0.8)
Papua New Guinea (PNG) 2016	DHS	1815	59.5 (55.4-63.4)	27.4 (24.5-30.6)	36.1 (32.2-40.2)	1.9 (1.1-3.1)
Timor Leste (TLS) 2016	DHS	1423	93.1 (91.3-94.6)	68.6 (65.6-71.4)	21.6 (18.8-24.7)	0.3 (0.1-0.8)
Tonga (TON) 2019	MICS	246	99.5 (97.8-99.9)	95.6 (92.0-97.6)	3.6 (1.4-8.8)	0.0 (0.0-0.1)
Tuvalu (TUV) 2019	MICS	114	93.4 (86.3-97.0)	80.5 (71.0-87.5)	1.7 (0.4-7.2)	1.7 (0.4-7.2)
Vietnam (VNM) 2020	MICS	860	98.3 (97.4-98.9)	94.2 (92.3-95.6)	4.9 (3.3-7.1)	0.0 (0.0-0.1)
Samoa (WSM) 2019	MICS	584	93.3 (90.0-95.5)	84.4 (80.3-87.7)	29.5 (25-34.5)	21.9 (17.8-26.6)
**Eastern & Southern Africa**		**31669**	**71.8 (70.7-72.9)**	**45.7 (44.6-46.8)**	**13.8 (13.0-14.7)**	**1.4 (1.2-1.6)**
Angola (AGO) 2015	DHS	2845	63.3 (60.7-65.8)	40.5 (37.6-43.4)	31.2 (28.6-34.0)	1.6 (1.0-2.6)
Burundi (BDI) 2016	DHS	2596	49.5 (47.1-52.0)	20.3 (18.4-22.3)	0.8 (0.5-1.2)	0.0 (0.0-0.2)
Comoros (COM) 2022	MICS	857	91.3 (88.7-93.3)	69.0 (64.7-73.0)	19.6 (16.2-23.6)	13.4 (10.8-16.5)
Ethiopia (ETH) 2016	DHS	2937	57.0 (53.3-60.6)	16.8 (14.1-19.9)	25.7 (22.8-28.9)	0.9 (0.5-1.6)
Kenya (KEN) 2022	DHS	3679	94.7 (93.7-95.5)	79.2 (77.3-80.9)	2.9 (2.3-3.6)	0.2 (0.1-0.5)
Lesotho (LSO) 2018	MICS	667	94.0 (91.4-95.8)	85.7 (82.2-88.6)	8.5 (6.3-11.2)	4.0 (2.4-6.3)
Madagascar (MDG) 2021	DHS	2345	47.1 (44.3-49.9)	26.1 (23.6-28.6)	21.7 (19.4-24.3)	0.3 (0.1-0.9)
Mozambique (MOZ) 2022	DHS	1727	68.6 (65.4-71.6)	32.8 (30.1-35.7)	23.9 (20.9-27.3)	3.9 (2.9-5.4)
Malawi (MWI) 2019	MICS	3206	52.1 (49.4-54.7)	27.5 (25.4-29.8)	4.6 (3.7-5.7)	3.5 (2.7-4.5)
Rwanda (RWA) 2019	DHS	1572	72.8 (70.3-75.2)	43.6 (40.6-46.6)	0.4 (0.2-0.9)	0.0 (0.0-0.0)
Eswatini (SWZ) 2021	MICS	422	97.7 (95.6-98.8)	90.3 (84.2-94.2)	6.3 (4.3-9.4)	4.2 (2.5-6.8)
Tanzania (TZA) 2022	DHS	2143	84.8 (82.7-86.8)	56.0 (52.7-59.3)	5.4 (4.2-6.9)	0.3 (0.1-0.8)
Uganda (UGA) 2016	DHS	2922	77.0 (75.0-79.0)	41.5 (38.9-44.2)	5.1 (4.1-6.3)	0.5 (0.2-0.9)
South Africa (ZAF) 2016	DHS	670	97.3 (94.9-98.5)	89.5 (85.9-92.3)	8.8 (6.4-12.2)	4.1 (2.5-6.6)
Zambia (ZMB) 2018	DHS	1928	72.8 (70.1-75.3)	44.8 (41.5-48.3)	2.1 (1.4-3.1)	0.3 (0.1-0.8)
Zimbabwe (ZWE) 2019	MICS	1153	86.9 (84.3-89.1)	66.8 (63.6-69.9)	5.5 (3.9-7.6)	5.3 (3.7-7.4)
**Eastern Europe & Central Asia**		**3072**	**98.5 (98.0-98.9)**	**66.1 (63.2-68.9)**	**6.0 (5.0-7.1)**	**0.5 (0.4-0.8)**
Armenia (ARM) 2015	DHS	345	99.7 (98.1-100.0)	97.4 (95.1-98.6)	1.5 (0.7-3.3)	0.0 (0.0-0.0)
Kyrgyzstan (KGZ) 2018	MICS	643	99.4 (98.4-99.8)	91.6 (88.0-94.1)	9.4 (6.7-12.9)	0.0 (0.0-0.0)
Tajikistan (TJK) 2017	DHS	1297	97.3 (96.1-98.2)	48.6 (44.7-52.5)	7.6 (6.1-9.4)	1.0 (0.7-1.6)
Turkmenistan (TKM) 2015	MICS	787	99.6 (98.7-99.8)	41.0 (24.9-59.3)	0.7 (0.3-1.5)	0.3 (0.1-1.1)
**Latin America & Caribbean**		**5317**	**82.7 (80.7-84.6)**	**65.9 (63.8-68.1)**	**10.8 (9.3-12.6)**	**2.8 (2.3-3.5)**
Cuba (CUB) 2019	MICS	1119	81.7 (75.2-86.9)	73.7 (68.4-78.5)	2.6 (1.3-5.0)	1.3 (0.5-3.5)
Dominican Republic (DOM) 2019	MICS	1674	93.4 (91.5-94.9)	86.6 (84.2-88.6)	8.1 (6.5-9.9)	6.3 (5.0-7.9)
Guyana (GUY) 2019	MICS	565	86.4 (77.9-92.0)	82.2 (74.2-88.1)	8.8 (6.2-12.3)	8.8 (5.5-11.4)
Haiti (HTI) 2016	DHS	1196	73.8 (70.4-77.0)	44.7 (41.2-48.3)	16.5 (13.3-20.1)	0.1 (0.0-0.4)
Suriname (SUR) 2018	MICS	763	96.7 (94.7-98.0)	92.9 (89.3-95.3)	19.7 (15.5-24.7)	13.0 (9.9-17.0)
**Middle East & North Africa**		**13830**	**95.2 (94.5-95.8)**	**79.8 (78.4-81.2)**	**14.1 (13.1-15.1)**	**11.1 (10.2-12.0)**
Algeria (DZA) 2018	MICS	2953	94.2 (92.8-95.4)	87.7 (85.9-89.3)	4.5 (3.7-5.5)	3.0 (2.3-3.8)
Iraq (IRQ) 2018	MICS	3205	98.9 (98.2-99.3)	71.5 (68.2-74.5)	13.3 (11.4-15.5)	8.8 (7.1-10.8)
Jordan (JOR) 2017	DHS	1945	98.5 (97.5-99.1)	91.1 (89.3-92.6)	7.4 (5.7-9.7)	0.1 (0.0-0.2)
State of Palestine (PSE) 2019	MICS	1330	97.7 (96.6-98.4)	87.9 (84.7-90.6)	4.5 (3.5-5.8)	1.6 (1.0-2.5)
Tunisia (TUN) 2018	MICS	656	97.2 (95.3-98.3)	90.9 (88.0-93.2)	4.6 (3.1-6.7)	1.3 (0.7-2.5)
Yemen (YEM) 2022	MICS	3741	89.8 (87.8-91.5)	52.6 (46.6-58.6)	32.5 (30.2-34.9)	30.9 (28.6-33.3)
**South Asia**		**54763**	**95.7 (95.4-95.9)**	**50.0 (48.3-51.7)**	**8.1 (7.6-8.7)**	**1.2 (1.1-1.4)**

Afghanistan (AFG) 2022	MICS	6177	85.5 (83.7-87.0)	37.9 (35.8-40.1)	35.7 (33.4-38.1)	31.9 (29.6-34.3)
Bangladesh (BGD) 2017	DHS	1666	96.4 (95.1-97.3)	61.9 (59.1-64.7)	1.5 (0.9-2.5)	0.0 (0.0-0.0)
India (IND) 2019	DHS	43436	96.1 (95.8-96.3)	58.2 (56.5-60.0)	6.4 (6.0-6.7)	0.2 (0.2-0.3)
Maldives (MDV) 2016	DHS	590	99.9 (99.6-100)	98.3 (97.2-99.0)	9.2 (6.6-12.8)	0.5 (0.2-1.2)
Nepal (NPL) 2022	DHS	1001	97 (95.5-98.0)	79.0 (75.5-82.1)	5.3 (3.8-7.3)	0.3 (0.1-0.9)
Pakistan (PAK) 2017	DHS	1893	95.6 (94.3-96.6)	38.3 (34.9-41.9)	13.7 (11.2-16.5)	0.2 (0.1-0.5)
**West & Central Africa**		**37464**	**79.0 (77.7-80.1)**	**51.0 (49.7-52.3)**	**24.7 (23.5-25.9)**	**17.6 (16.5-18.7)**
Benin (BEN) 2021	MICS	2539	86.2 (84.3-87.9)	53.7 (50.8-56.6)	19.5 (17.4-21.8)	13.0 (11.4-14.9)
Burkina Faso (BFA) 2021	DHS	2313	97.5 (96.6-98.1)	76.3 (74.1-78.3)	5.1 (4.0-6.4)	0.6 (0.3-1.1)
CAR (CAF) 2018	MICS	1688	32.8 (29.8-36.0)	13.5 (11.8-15.3)	45.0 (41.7-48.3)	41.0 (37.6-44.4)
Cote d'Ivoire (CIV) 2021	DHS	1920	94.6 (93.1-95.8)	75.2 (72.6-77.7)	30.0 (26.5-33.9)	0.7 (0.4-1.6)
Cameroon (CMR) 2018	DHS	1824	86.0 (83.8-87.9)	58.5 (55.2-61.8)	16.7 (14.2-19.5)	0.7 (0.4-1.2)
Democratic Rep Congo (COD) 2017	MICS	4250	45.9 (42.2-49.7)	29.9 (26.6-33.6)	34.1 (30.2-38.2)	32.1 (28.4-36.1)
Gabon (GAB) 2019	DHS	1271	97.0 (95.6-98.0)	89.6 (86.8-91.8)	16.4 (13.2-20.1)	2.0 (1.0-4.2)
Ghana (GHA) 2022	DHS	1973	94.7 (92.9-96.1)	76.3 (73.6-78.8)	2.9 (2.1-4.2)	0.2 (0.0-0.8)
Guinea (GIN) 2018	DHS	1408	91.1 (89.1-92.8)	70.1 (67.1-72.8)	37.7 (34.1-41.4)	0.8 (0.3-1.7)
Guinea Bissau (GNB) 2018	MICS	1409	95.4 (93.2-97.0)	50.7 (46.9-54.6)	7.0 (5.2-9.4)	6.0 (4.3-8.3)
Gambia (GMB) 2019	DHS	1582	98.8 (98.0-99.2)	75.2 (72.2-77.9)	1.7 (0.9-3.0)	0.2 (0.0-0.9)
Liberia (LBR) 2019	DHS	1063	66.5 (62.0-70.7)	35.4 (31.0-40.1)	8.6 (6.3-11.5)	0.2 (0.1-0.7)
Mali (MLI) 2018	DHS	1946	91.2 (89.3-92.8)	51.1 (47.5-54.6)	17.9 (15.1-21.1)	1.0 (0.5-1.8)
Mauritania (MRT) 2019	DHS	2119	91.3 (89.2-93.0)	77.6 (74.7-80.3)	12.2 (10.1-14.6)	1.0 (0.6-1.6)
Niger (NER) 2021	DHS	1619	76.3 (72.8-79.5)	31.9 (28.0-36.0)	19.4 (15.8-23.6)	0.4 (0.2-0.8)
Nigeria (NGA) 2021	MICS	5582	84.9 (83.4-86.3)	51.8 (49.2-54.5)	29.7 (27.5-32.0)	27.2 (25.1-29.4)
Senegal (SEN) 2019	DHS	1183	98.4 (97.3-99.1)	66.1 (62.2-69.8)	3.8 (2.6-5.6)	0.0 (0.0-0.0)
Sierra Leone (SLE) 2019	DHS	1861	71.4 (68.5-74.1)	33.8 (31.3-36.5)	5.4 (4.2-6.9)	0.6 (0.3-1.3)
São Tome and Principe (STP) 2019	MICS	349	86.5 (81.7-90.2)	64.8 (58.8-70.4)	2.3 (1.1-4.7)	2.1 (0.9-4.5)
Togo (TGO) 2017	MICS	973	87.0 (83.9-89.5)	50.7 (45.7-55.8)	9.2 (6.8-12.2)	7.1 (5.0-10.0)
**Pooled prevalence**		**163,527**	**87.2 (86.9-87.5)**	**56.2 (55.6-56.8)**	**13.4 (13.0-13.8)**	**5.6 (5.3-5.8)**

95% CI Confidence Interval

Notes:

1. Unweighted value.
2. Missing values were treated as unvaccinated children.
3. Missing values for *mother own mobile* in Afghanistan (n=70), Algeria (n=15), Benin (n=48), CAR (n=68), Comoros (n=40), Congo Democratic Republic (n=24), Cuba (n=9), Dominican Republic (n=57), Eswatini (n=264), Ethiopia (n=1008), Fiji (n=17), Guinea Bissau (n=28), Guyana (n=21), India (n=36861), Iraq (n=11), Kiribati (n=23), Kyrgyzstan (n=20), Lao (n=81), Lesotho (n=97), Malawi (n=78), Mongolia (n=42), Nigeria (n=909), Samoa (n=40), São Tomé and Principe (n=13), Suriname (n=62), Togo (n=17), Tonga (n=29), Tunisia (n=7), Turkmenistan (n=747), Tuvalu (n=16), Vietnam (n=33), Yemen (n=2554), and Zimbabwe (n=55). There are differential losses, comparing the total sample with those with information for *mother own mobile according to wealth quintiles*, in Yemen, Nigeria and Turkmenistan, with higher proportion of children in the wealthiest quintiles in the sample evaluating *mother own mobile.*

Table S2. Zero-dose prevalence according to mothers' and household mobile ownership, by residence and wealth quintiles.

	Zero-DPT prevalence		
	All children *% (95% CI)*	*Household owns a mobile* *% (95% CI)*	*Mother owns a mobile* *% (95% CI)*
**Area of residence**			
Urban	9.7 (9.1-10.3)	9.2 (8.7 – 9.8)	8.4 (7.8 – 9.1)
Rural	15.4 (15.0-16.0)	13.8 (13.3 – 14.3)	12.3 (11.6 – 13.1)
**Wealth quintiles**			
Poorest	20.7 (19.8-21.6)	19.3 (18.4-20.3)	18.5 (17.2 – 19.8)
Second	15.5 (14.8-16.3)	14.8 (14.1 – 15.6)	13.9 (12.9 – 15.0)
Third	12.4 (11.7 – 13.1)	12.0 (11.3 – 12.7)	11.4 (10.5 – 12.3)
Fourth	9.2 (8.5 – 9.8)	9.0 (8.3 – 9.7)	8.5 (7.7 – 9.3)
Wealthiest	6.8 (6.2 – 7.4)	6.7 (6.1 – 7.3)	6.6 (5.9 – 7.3)
**All children**	**13.4 (13.0-13.8)**	**12.0 (11.6-12.4)**	**10.3 (9.7-10.8)**

95% CI – 95% Confidence Interval

Table S3. Simulation analyses of zero-dose prevalence (national, by wealth quintiles, and by place of residence) of the potential impact of promoting immunizations through mobile phones under two effectiveness scenarios.

**Contact through mother's mobile phone**						
	All countries % (95% CI)		*High national zero-dose prevalence and high mobile coverage* % (95% CI)		*High national zero-dose prevalence and low mobile coverage* % (95% CI)	
	0% effectiveness	100% effectiveness	0% effectiveness	100% effectiveness	0% effectiveness	100% effectiveness
**All children**	13.4 (13.0 – 13.8)	9.7 (9.3-10.0)	30.8 (28.6-33.1)	9.8 (8.7 – 11.0)	23.9 (22.9 – 24.9)	18.9 (18.0 – 20.0)
**Area of residence**						
Urban	9.7 (9.1 – 10.2)	5.0 (4.6 – 5.4)	28.4 (24.3 – 32.6)	5.0 (3.3 – 6.7)	12.7 (11.4 – 14.1)	7.2 (6.2 – 8.2)
Rural	15.4 (14.9 – 16.0)	12.0 (11.6 – 12.5)	32.6 (30.2 – 35.0)	13.2 (11.6 – 14.8)	29.2 (27.8 – 30.5)	24.4 (23.2 – 25.7)
**Wealth quintiles**						
Poorest	20.7 (19.8 – 21.6)	17.7 (16.9 – 18.5)	39.6 (35.6 – 43.7)	13.4 (10.7 – 16.1)	40.0 (37.4 – 42.3)	36.7 (34.3 – 39.1)
Second	15.5 (14.8 – 16.3)	12.0 (11.3 – 12.6)	30.5 (27.0 – 34.1)	5.5 (3.6 – 7.4)	29.2 (27.2 – 31.2)	24.0 (22.3 – 25.7)
Third	12.4 (11.7 – 13.1)	8.2 (7.6 – 8.8)	29.1 (25.2 – 33.1)	4.0 (2.0 – 6.0)	22.1 (20.3 – 23.8)	16.1 (14.6 – 17.7)
Fourth	9.2 (8.5 – 9.8)	5.1 (4.6 – 5.5)	26.2 (20.3 – 32.1)	0.9 (0.5 – 1.4)	14.6 (13.0 – 16.1)	8.5 (7.4 – 9.6)
Wealthiest	6.8 (6.2 – 7.4)	2.8 (2.4 – 3.2)	26.6 (19.1 – 34.2)	0.3 (-0.0 – 0.6)	8.2 (7.0 – 9.4)	3.5 (2.7 – 4.3)
**Contact through household's mobile phone**						
	All countries % (95% CI)		*High national zero-dose prevalence and high mobile coverage* % (95% CI)		*High national zero-dose prevalence and low mobile coverage* % (95% CI)	
	0% effectiveness	100% effectiveness	0% effectiveness	100% effectiveness	0% effectiveness	100% effectiveness
**All children**	13.4 (13.0 – 13.8)	9.7 (9.3-10.0)	30.8 (28.6-33.1)	2.4 (1.9 – 2.9)	27.3 (24.9 – 29.7)	7.2 (6.7 – 7.7)
**Area of residence**						
Urban	9.7 (9.1 – 10.2)	5.0 (4.6 – 5.4)	28.4 (24.3 – 32.6)	0.8 (0.3 – 1.4)	11.4 (8.4 – 14.3)	1.4 (1.1 – 1.7)
Rural	15.4 (14.9 – 16.0)	12.0 (11.6 – 12.5)	32.6 (30.2 – 35.0)	3.5 (2.7 – 4.2)	32.9 (30.1 – 35.8)	10.1 (9.4 – 10.8)
**Wealth quintiles**						
Poorest	20.7 (19.8 – 21.6)	17.7 (16.9 – 18.5)	39.6 (35.6 – 43.7)	6.8 (5.3 – 8.4)	42.2 (36.4 – 48.1)	19.5 (17.9 – 21.0)
Second	15.5 (14.8 – 16.3)	12.0 (11.3 – 12.6)	30.5 (27.0 – 34.1)	2.5 (1.3 – 3.8)	31.3 (26.7 – 36.0)	7.7 (6.8 – 8.7)
Third	12.4 (11.7 – 13.1)	8.2 (7.6 – 8.8)	29.1 (25.2 – 33.1)	1.3 (0.5 – 2.0)	26.8 (22.4 – 31.2)	3.8 (3.2 – 4.5)
Fourth	9.2 (8.5 – 9.8)	5.1 (4.6 – 5.5)	26.2 (20.3 – 32.1)	0.1 (0.0 – 0.2)	15.7 (11.8 – 19.6)	1.1 (0.8 – 1.4)
Wealthiest	6.8 (6.2 – 7.4)	2.8 (2.4 – 3.2)	26.6 (19.1 – 34.2)	0.1 (0.0 – 0.4)	12.1 (8.0 – 16.2)	0.2 (0.1 – 0.4)

95% CI – 95% Confidence Interval

Table S4. Distribution of mothers according to social independence and decision-making domains of the women's empowerment index (Ewerling et al. 2021) (SWPER)

*SWPER domains**	*All mothers* *% (95% CI)*	*Mother with their own mobile phone* *% (95% CI)*	*Household owns a mobile phone* *% (95% CI)*
**Social independence domain**			
Low empowerment	33.0 (32.2-33.7)	36.0 (34.8-37.3)	81.8 (80.9-82.6)
Medium empowerment	33.2 (32.5-33.9)	49.9 (48.6-51.1)	86.1 (85.3-86.9)
High empowerment	33.8 (33.0-34.6)	71.7 (70.4-73.0)	92.0 (91.3-92.7)
**Decision-making domain**			
Low empowerment	24.2 (23.5-24.9)	41.1 (39.5-42.8)	84.2 (83.1-85.2)
Medium empowerment	25.7 (25.0-26.4)	51.6 (50.3-52.9)	86.2 (85.4-87.0)
High empowerment	50.1 (49.4-50.8)	59.2 (58.0-60.4)	86.9 (86.3-87.6)

*The survey-based indicator of women's empowerment (SWPER) is comprised by three domains: attitude to violence, social independence and decision making. Social independence is based on six items: frequency of reading a newspaper or magazine; years of completed schooling; difference in years of schooling between the woman and her partner; age difference between the woman and her partner; age at first cohabitation; and age at first birth (imputed if nulliparous). Decision making domain is comprised of three questions on who makes decisions in the household in regard to the respondent's health care, major expenses and to visits to family and relatives (Ewerling et *al*., 2021).

** Data from 34 countries with information on mobile phone ownership and women's empowerment.

Ewerling, F., F. C. Wehrmeister, C. G. Victora, A. Raj, L. McDougal, and A. J. D. Barros. 2021. "Is Women's Empowerment Associated with Coverage of RMNCH Interventions in Low- and Middle-Income Countries? An Analysis Using a Survey-Based Empowerment Indicator, the SWPER." *J Glob Health* 11 (04015). https://doi.org/10.7189/jogh.11.04015.

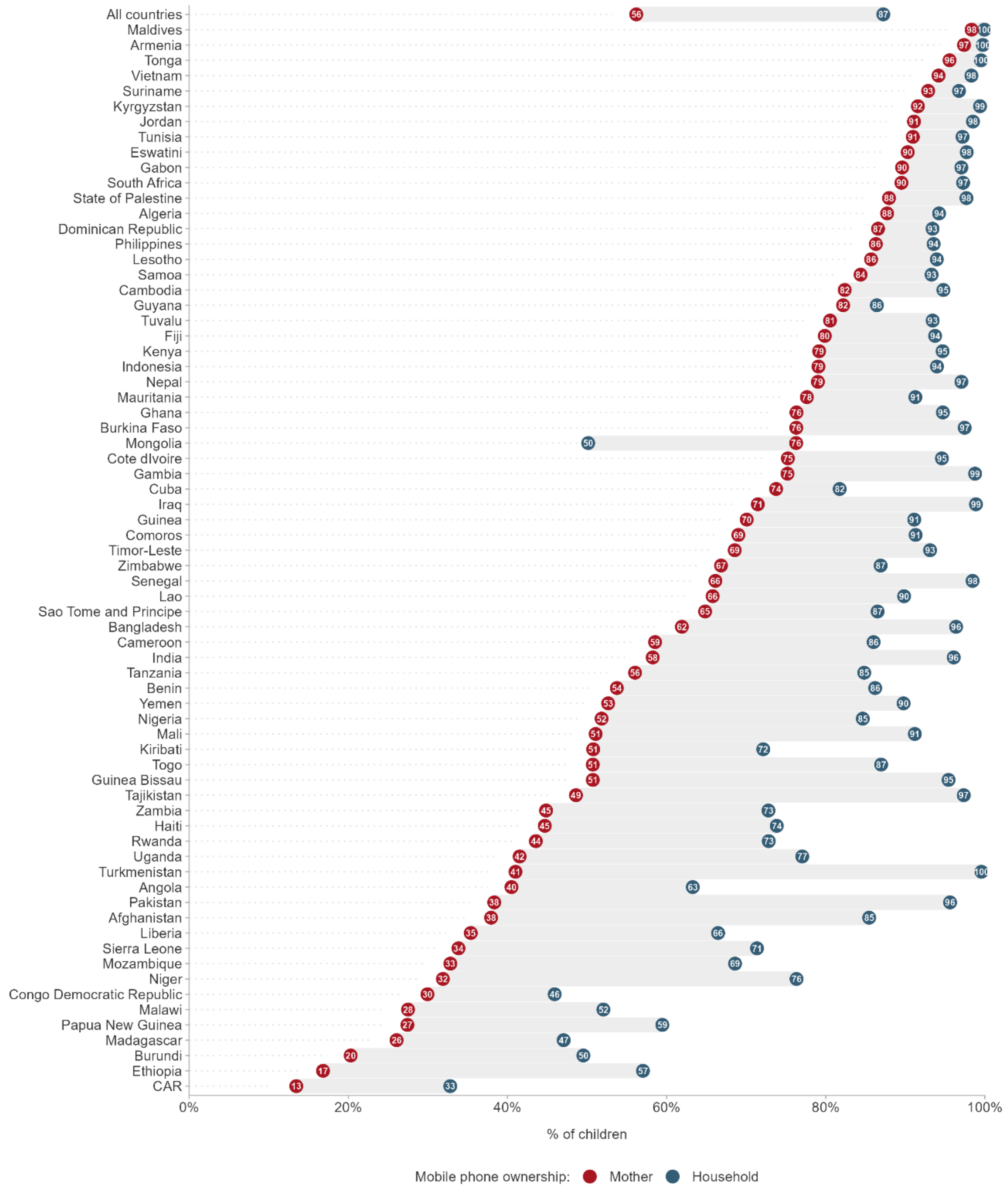

Figure S1. Ownership of mobile phones by households and by mothers in 70 countries.

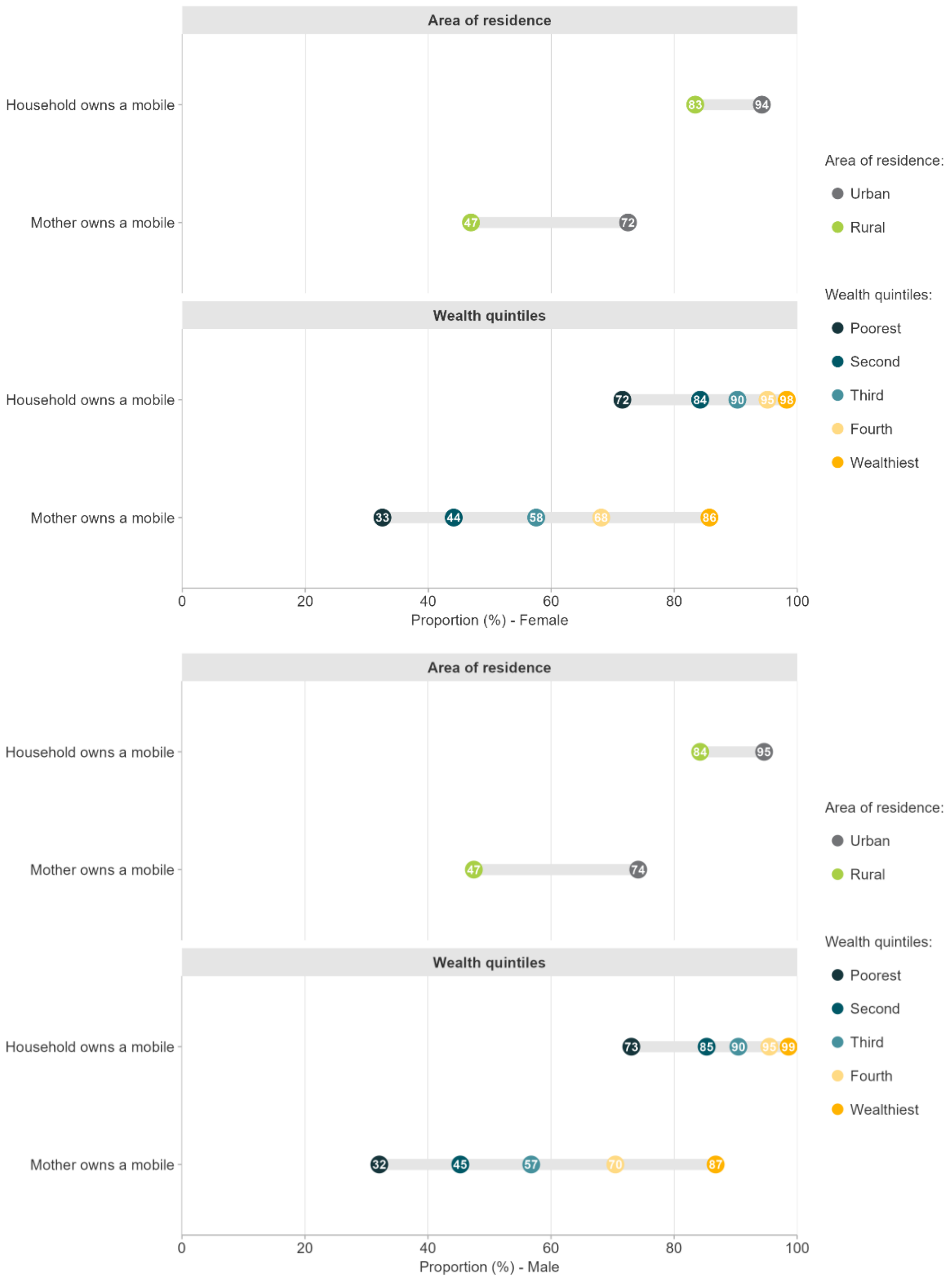

Figure S2. Proportion of households and mothers with a mobile phone according to wealth quintiles and area of residence according to sex of the child. Pooled analyses of 70 countries.

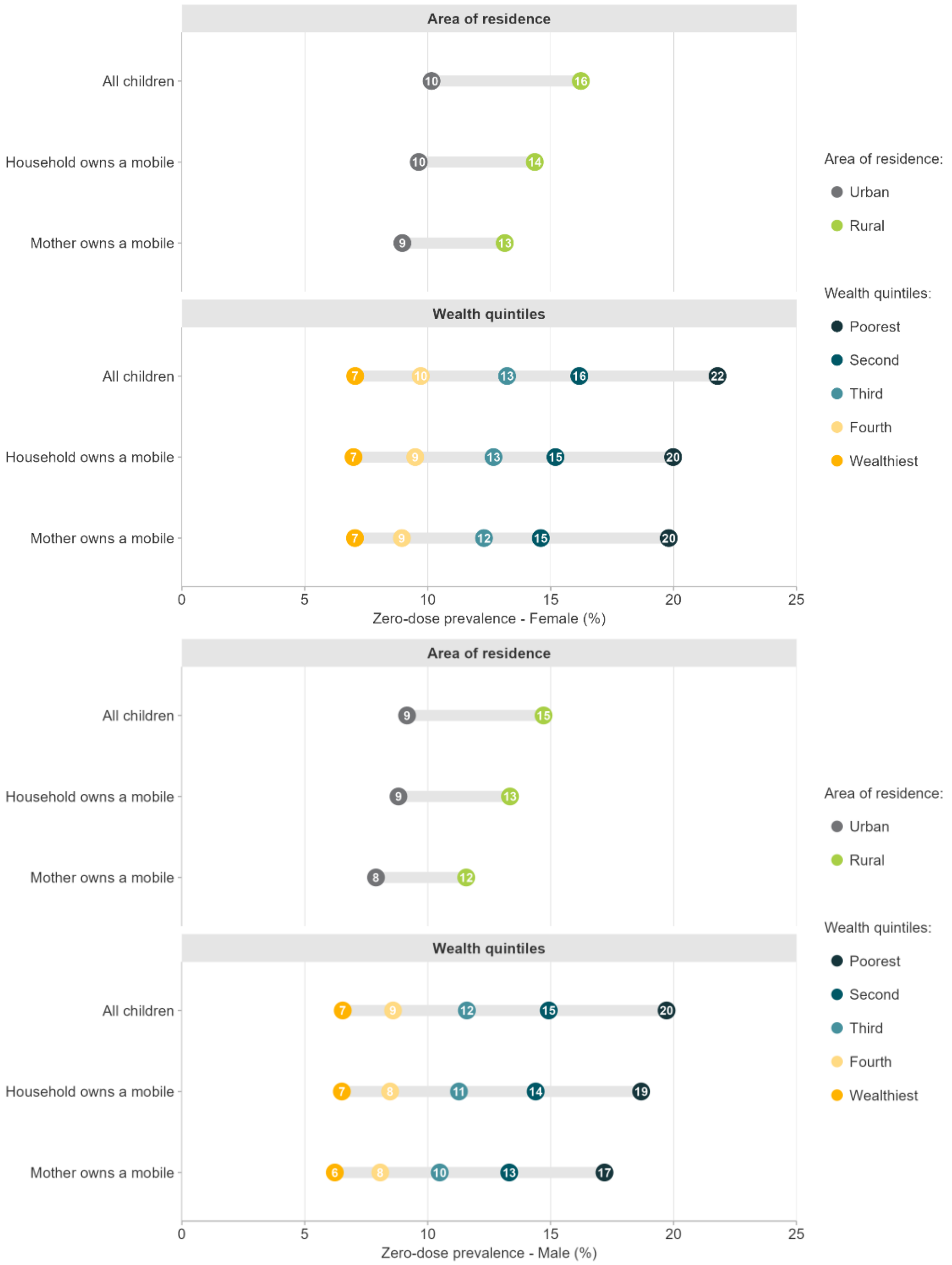

Figure S3. Zero-dose prevalence according to place of residence and wealth quintiles for all children, for children in households with a mobile phone, and for children whose mother owns a mobile phone according to sex. Pooled analyses of 70 countries. All P-values <0.001.

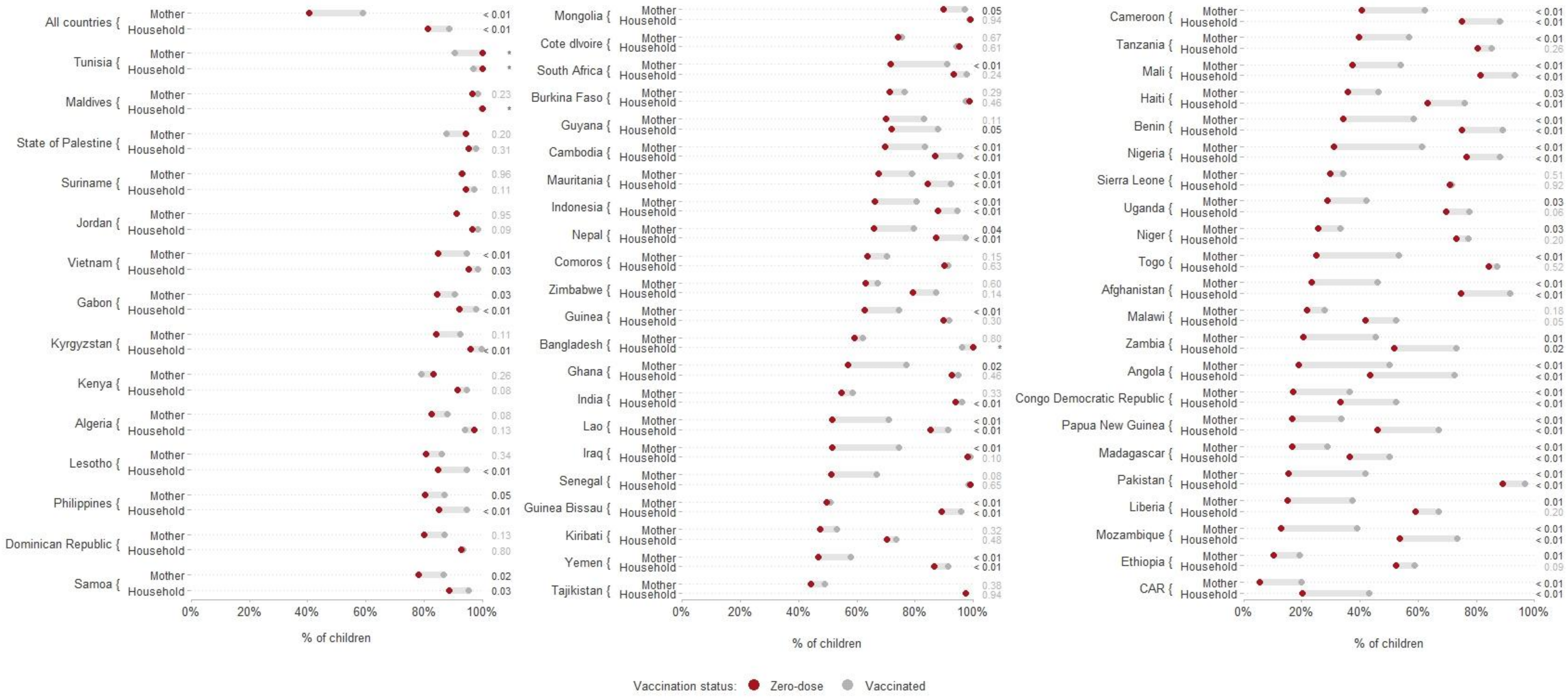

Figure S4. Proportions of households and mothers with a mobile phone among zero-dose and vaccinated children. Countries are ranked by the percentages of mobile phone ownership by mothers. P values are shown on the right side of each bar. (*) Test could not be performed because of 100% ownership in both groups.

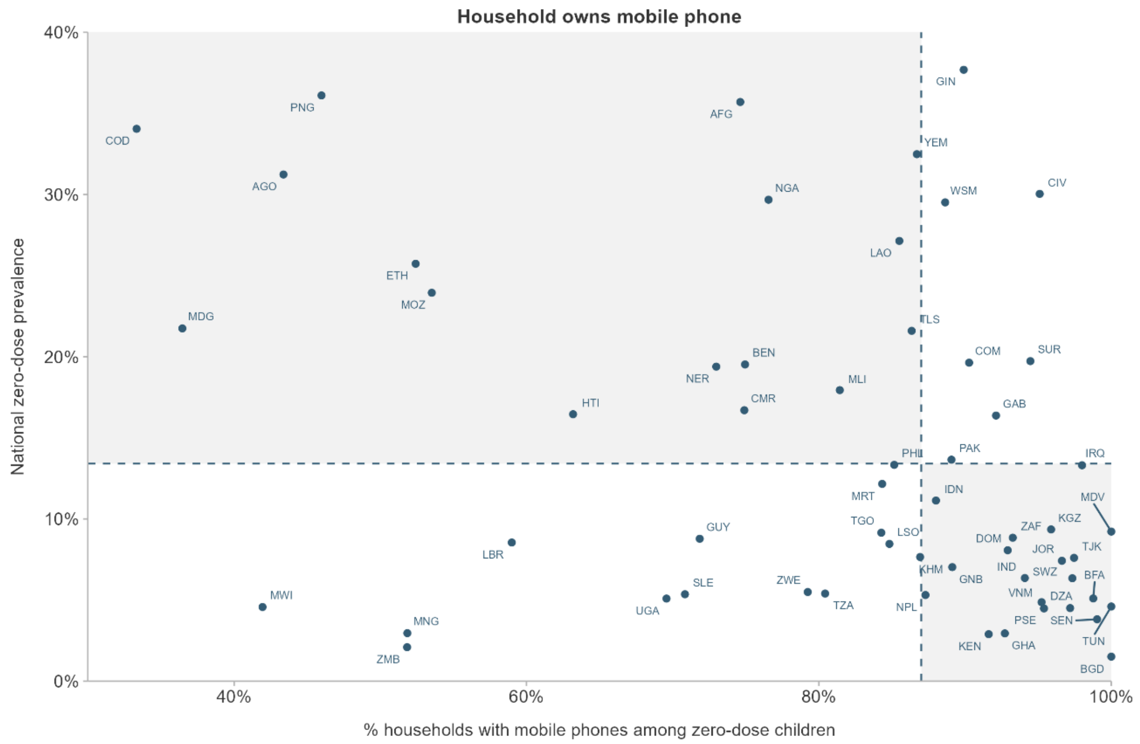

Figure S5. National zero-dose prevalence according to the proportions of households with zero-dose children in which a mobile phone is available. Dashed lines show the median values of the axes. Pearson correlation coefficient = -0.44, 95% CI -0.63; -0.21.

Checklist S1. STROBE Statement—checklist of items that should be included in reports of observational studies

	Item No.	Recommendation	Relevant text from manuscript
**Title and abstract**	1	(*a*) Indicate the study's design with a commonly used term in the title or the abstract	"Can unvaccinated children be reached through mobile phones?<br><br>Analyses of national cross-sectional surveys from 70 countries"
		(*b*) Provide in the abstract an informative and balanced summary of what was done and what was found	"Methods: We analysed 70 nationally representative surveys with data on immunizations and mobile phone ownership by households and mothers and correlated ownership with household wealth and place of residence (…)"<br><br>"Results: The surveys included 163,527 children aged 12-23 months, with 13.4% being zero-dose. Among them, 34% of mothers and 73% of households had a mobile phone, compared to 60% and 89% for vaccinated children (…)"<br><br>"Conclusions: The potential impact of mHealth for reaching zero-dose children may be limited by mobile phone ownership among mothers and families, particularly among the poor, where unvaccinated children are concentrated."
**Introduction**			
Background/rationale	2	Explain the scientific background and rationale for the investigation being reported	"(…)A recent umbrella review[14] examined 62 systematic reviews on multiple types of child immunization interventions and found that caregiver-oriented strategies, such as sending written and pictorial messages via Short Message Service (SMS) or flyers, led to a pooled improvement of 24% in coverage rates. However, the vast majority of the studies identified by the reviewers focused on follow-up reminders for parents of children late in receiving their shots. The review only found four studies on zero-dose children, which showed inconclusive results. (…)"
Objectives	3	State specific objectives, including any prespecified hypotheses	"The present analyses examine the likelihood that mHealth interventions could help reach zero-dose children with vaccination in 70 LMICs based on data on current vaccine coverage and ownership of mobile phones. First, we describe the proportions of mothers and households who own mobile phones in these countries and how these proportions vary by wealth and urban/rural residence. Second, we show how phone ownership varies according to the zero-dose status of the children. Lastly, we simulate the potential impact on zero-dose prevalence and related inequalities by (a) using cell phones to deliver

			messages to mothers and families of unvaccinated children and (b) assuming different scenarios of how effective such cell phone messages might be in leading these children to be vaccinated.”
**Methods**			
Study design	4	Present key elements of study design early in the paper	“The global database maintained by the International Center for Equity in Health (ICEH) includes almost 460 publicly available, nationally representative surveys from 120 countries (https://www.equidade.org/about-us).”
Setting	5	Describe the setting, locations, and relevant dates, including periods of recruitment, exposure, follow-up, and data collection	“The present analyses relied upon 70 Demographic and Health Surveys or DHS 16 and Multiple Indicator Cluster Surveys or MICS17 containing information on mobile phone ownership and child vaccinations.”
Participants	6	(*a*) *Cohort study*—Give the eligibility criteria, and the sources and methods of selection of participants. Describe methods of follow-up<br>*Case-control study*—Give the eligibility criteria, and the sources and methods of case ascertainment and control selection. Give the rationale for the choice of cases and controls<br>*Cross-sectional study*—Give the eligibility criteria, and the sources and methods of selection of participants	“(…) surveyed children aged 12-23 months (…)”<br>“When a home-based record with vaccination information was not available for inspection, the survey respondent’s report on vaccinations was recorded. Missing values were treated as a lack of vaccination.”
		(*b*) *Cohort study*—For matched studies, give matching criteria and number of exposed and unexposed<br>*Case-control study*—For matched studies, give matching criteria and the number of controls per case	(-)
Variables	7	Clearly define all outcomes, exposures, predictors, potential confounders, and effect modifiers. Give diagnostic criteria, if applicable	“The outcome was zero-dose prevalence, or the proportions of surveyed children aged 12-23 months who failed to receive any doses of a DPT-containing vaccine.”<br>“Information from the women’s and household DHS modules allowed for assessing mobile phones in the household and phones owned by children's mothers.”<br>“Children were also characterized according to household wealth quintiles and urban-rural residence.”

Data sources/ measurement	8	For each variable of interest, give sources of data and details of methods of assessment (measurement). Describe comparability of assessment methods if there is more than one group	"The outcome was zero-dose prevalence, or the proportions of surveyed children aged 12-23 months who failed to receive any doses of a DPT-containing vaccine[23]. When a home-based record with vaccination information was not available for inspection, the survey respondent's report on vaccinations was recorded[24]. Missing values were treated as a lack of vaccination as is standard practice in coverage analyses[24]. Table S1 in the Online Supplementary Document provides information by country on the sources of information on immunization status and on missing values. Information from the women's and household DHS questionnaires allowed for assessing mobile phones in the household and phones owned by children's mothers."<br><br>"Quintiles were based on wealth indices provided in the DHS datasets, which are derived through principal component analyses of household assets (presence of radio, television, refrigerator, etc.) and characteristics of the homes, including the availability of electricity, water supply, and sanitary facilities[25]. Since relevant assets may differ between urban and rural households, the wealth index is initially calculated separately for each area and later combined into a single national index by using a scaling procedure to ensure comparability between the area scores. The area of residence is categorized as urban or rural, depending on country-specific delimitations[25]."
Bias	9	Describe any efforts to address potential sources of bias	"Since relevant assets may differ between urban and rural households, the wealth index is initially calculated separately for each area and later combined into a single national index by using a scaling procedure to ensure comparability between the area scores."<br><br>"(…) accounted for the multi-stage survey design"
Study size	10	Explain how the study size was arrived at	(-)

Continued on next page

Quantitative variables	11	Explain how quantitative variables were handled in the analyses. If applicable, describe which groupings were chosen and why	"The analyses included an examination of mobile phone ownership by wealth and residence, followed by zero-dose prevalence by mobile phone ownership, wealth, and residence. Statistical analyses were carried out at the individual level, using R version 4.3.1 and Stata version 1821, and accounted for the multi-stage survey design."
Statistical methods	12	(*a*) Describe all statistical methods, including those used to control for confounding	"The analyses included an examination of mobile phone ownership by wealth and residence, followed by zero-dose prevalence by mobile phone ownership, wealth, and residence. Statistical analyses were carried out at the individual level, using R version 4.3.1 and Stata version 18[26], and accounted for the multi-stage survey design. In the pooled analysis of the 70 countries, zero-dose prevalence according to explanatory variables was calculated using logistic regression and the "margins" command in Stata, including fixed effects for each country. The pooled estimates were weighted by national populations of children aged 12-23 months in 2019 (the median year of the surveys).<br><br>Simulation analyses were conducted to estimate the potential impact on zero-dose prevalence of digital interventions using mobile phones under five hypothetical impact scenarios: 0%, 10%, 25%, 50%, and a hypothetical 100% effectiveness levels. The first (0%) scenario indicated that the intervention had no effect. In contrast, in the last (100%) scenario, the intervention would result in every contact through a mobile phone, leading to the child being immunized. National post-intervention zero-dose prevalence was estimated from baseline national zero-dose prevalence minus the product of postulated intervention effectiveness times the proportion of zero-dose children with a mobile phone in the household, considering the intermediate scenarios (10%, 25%, and 50% effectiveness). The simulation exercise was repeated for children whose mothers had a mobile phone. Simulations were also carried out at the aggregate level for each subpopulation – wealth quintiles and urban/rural."
		(*b*) Describe any methods used to examine subgroups and interactions	"The simulation exercise was repeated for children whose mothers had a mobile phone. Simulations were also carried out at the aggregate level for each subpopulation – wealth quintiles and urban/rural."
		(*c*) Explain how missing data were addressed	"Missing values were treated as a lack of vaccination."
		(*d*) *Cohort study*—If applicable, explain how loss to follow-up was addressed<br><br>*Case-control study*—If applicable, explain how matching of cases and controls was addressed	"(…) accounted for the multi-stage survey design"

		Cross-sectional study—If applicable, describe analytical methods taking account of sampling strategy	
		(*e*) Describe any sensitivity analyses	(-)
**Results**			
Participants	13	(a) Report numbers of individuals at each stage of study—eg numbers potentially eligible, examined for eligibility, confirmed eligible, included in the study, completing follow-up, and analysed	"The total sample comprised 163,527 children from 70 countries, ranging from 114 children in Tuvalu to 43,436 in India (median of 1,600 [Interquartile Range 860- 2,215])."
		(b) Give reasons for non-participation at each stage	(-)
		(c) Consider use of a flow diagram	(-)
Descriptive data	14	(a) Give characteristics of study participants (eg demographic, clinical, social) and information on exposures and potential confounders	"Table S1 in the Online Supplementary Document describes the list of countries, the prevalence of mobile phone ownership by households and mothers, zero-dose prevalence, and the proportion of missing values on receipt of a DPT-containing vaccine (…)"
		(b) Indicate number of participants with missing data for each variable of interest	Table S1 in the Online Supplementary Document
		(c) *Cohort study*—Summarise follow-up time (eg, average and total amount)	(-)
Outcome data	15	*Cohort study*—Report numbers of outcome events or summary measures over time	(-)
		Case-control study—Report numbers in each exposure category, or summary measures of exposure	(-)
		Cross-sectional study—Report numbers of outcome events or summary measures	"In the pooled individual-level analyses of the 70 countries, the availability of mobile phones varied markedly according to wealth and place of residence (Figure 1). In the poorest quintile, 32% of mothers and 72% of households had a phone, compared to 86% and 98%, respectively, in the wealthiest quintile. Ownership was 47% among rural and 73% among urban mothers, whereas the corresponding gap for household phones was from 84% to 94%. These results did not vary according to sex of the child (Figure S2 in the Online Supplementary Document).

			Zero-dose prevalence in the pooled analyses was 13.4% (95% CI = 13.0%-13.8%). The highest prevalence was observed when neither the mother nor the household had a mobile phone (25.3%; 95% CI = 23.9%-26.8%), followed by households with a phone where the mother did not have her own phone (19.2%; 95% CI = 18.3%-20.0%). The lowest prevalence was observed when mothers had their own phones (10.0%; 95% CI = 9.5%-10.6%). Adjustment for residence and wealth made little difference to these findings."<br><br>**AND** Table 1
Main results	16	(*a*) Give unadjusted estimates and, if applicable, confounder-adjusted estimates and their precision (eg, 95% confidence interval). Make clear which confounders were adjusted for and why they were included	"Pooled zero-dose prevalence by residence and wealth are shown in Figure 2 and Table S2 in the Online Supplementary Document. Prevalence was 5.7 percentage points (pp) higher in rural areas than in urban areas (…)"
		(*b*) Report category boundaries when continuous variables were categorized	(-)
		(*c*) If relevant, consider translating estimates of relative risk into absolute risk for a meaningful time period	(-)

Continued on next page

Other analyses	17	Report other analyses done—eg analyses of subgroups and interactions, and sensitivity analyses	(-)
**Discussion**			
Key results	18	Summarise key results with reference to study objectives	"Although mobile phones are increasingly common in LMICs, like access to immunization, their ownership is not equitably distributed. Our results confirmed earlier analyses showing that gender, wealth, and place of residence are strongly associated with the availability of such phones[11,27]. In our pooled analyses, 56% of the women had their own mobile, compared to 87% of the households. The finding that phones are available in almost nine out of ten households is impressive, but the pooled proportions ranged from 72% to 98%, from the poorest to the wealthiest quintile. The gap was wider for mothers' phones, ranging from 30% to 85%. The urban-rural gap was also marked, albeit narrower than the wealth-related gap. While gender gaps likely reflect cultural issues such as women's empowerment, socioeconomic position affects the ability to purchase a phone and to afford airtime, whereas place of residence may limit the availability of networks."
Limitations	19	Discuss limitations of the study, taking into account sources of potential bias or imprecision. Discuss both direction and magnitude of any potential bias	"Limitations include the cross-sectional nature of the data, that information was only available for 70 of 130 LMICs worldwide, and that the median date of these surveys was 2019. Based on a limited number of countries, it is estimated that the gender gap in mobile internet usage fell from 21 pp in 2019 to 15 pp in 2023, but still remains above 30 pp in Sub-Saharan Africa and South Asia[10]. The analyses of mobile phone ownership by wealth quintiles should be approached with caution, as mobile phones are part of the assets used to estimate the wealth index in many countries, which can create a tautological relationship. Despite this concern, the potential bias is likely minimal, as phones represent only one of 25-30 assets included in the wealth index. A study of 34 DHS and MICS found that mobile phones, on average, ranked 10th among the 27 or so assets frequently included in the index. The principal component analysis showed an average loading of 0.11 for mobile phones [Vidaletti and Barros, personal communication]. Thus, it is unlikely that such bias would explain the difference of over 50 percent points in woman's phone ownership between the wealthiest and poorest quintile."
Interpretation	20	Give a cautious overall interpretation of results considering objectives, limitations, multiplicity of analyses, results from similar studies, and other relevant evidence	"The strengths of our analyses include the national representativeness of the survey samples and the use of standardized questionnaires, indicator and variable definitions, and uniform statistical methods in the analyses. Ours was the largest multicountry analysis relating mobile phone ownership to sociodemographic and immunization patterns covering 70 of the approximately 130 LMICs.

			Limitations include the cross-sectional nature of the data, that information was only available for 70 of 130 LMICs worldwide, and that the median date of these surveys was 2019." (…)
Generalisability	21	Discuss the generalisability (external validity) of the study results	"Because unvaccinated children and their mothers are also less likely to visit health services for other types of care[13], they may not be included in facility registries even when a phone is available in the household. This suggests that strategies for reaching out to all available phones in a geographic area – such as SMS mass outreach campaigns – may achieve higher coverage than attempting to reach phones recorded in a health services registry."
**Other information**			
Funding	22	Give the source of funding and the role of the funders for the present study and, if applicable, for the original study on which the present article is based	"Authors TH, TM and DRH are employed by Gavi, the Vaccine Alliance, the study funder. As authors, they have participated in the study design, interpretation of results and writing up of the manuscript."

(-) not applicable
